# Glial activation is moderated by sex in response to amyloidosis but not to tau pathology in mouse models of neurodegenerative diseases

**DOI:** 10.1186/s12974-020-02046-2

**Published:** 2020-12-14

**Authors:** Gloria Biechele, Nicolai Franzmeier, Tanja Blume, Michael Ewers, Jose Medina Luque, Florian Eckenweber, Christian Sacher, Leonie Beyer, Francois Ruch-Rubinstein, Simon Lindner, Franz-Josef Gildehaus, Barbara von Ungern-Sternberg, Paul Cumming, Peter Bartenstein, Axel Rominger, Günter U. Höglinger, Jochen Herms, Matthias Brendel

**Affiliations:** 1 Department of Nuclear Medicine, University Hospital of Munich, LMU Munich, Marchioninstraße 15, 81377 Munich, Germany; 2 Institute for Stroke and Dementia Research, University Hospital of Munich, LMU Munich, Munich, Germany; 3 DZNE - German Center for Neurodegenerative Diseases, Munich, Germany; 4 Department of Nuclear Medicine, Inselspital, University Hospital Bern, Bern, Switzerland; 5 School of Psychology and Counselling, Queensland University of Technology, Brisbane, Australia; 6 Munich Cluster for Systems Neurology (SyNergy), Munich, Germany; 7 Department of Neurology, Hannover Medical School, Hannover, Germany; 8 Department of Neurology, Technical University Munich, Munich, Germany; 9 Center of Neuropathology and Prion Research, University of Munich, Munich, Germany

**Keywords:** Sex, Microglia, TSPO, Amyloid, Tau

## Abstract

**Background:**

In vivo assessment of neuroinflammation by 18-kDa translocator protein positron-emission-tomography (TSPO-PET) ligands receives growing interest in preclinical and clinical research of neurodegenerative disorders. Higher TSPO-PET binding as a surrogate for microglial activation in females has been reported for cognitively normal humans, but such effects have not yet been evaluated in rodent models of neurodegeneration and their controls. Thus, we aimed to investigate the impact of sex on microglial activation in amyloid and tau mouse models and wild-type controls.

**Methods:**

TSPO-PET (^18^F-GE-180) data of C57Bl/6 (wild-type), *App*^*NL-G-F*^ (β-amyloid model), and P301S (tau model) mice was assessed longitudinally between 2 and 12 months of age. The *App*^*NL-G-F*^ group also underwent longitudinal β-amyloid-PET imaging (Aβ-PET; ^18^F-florbetaben). PET results were confirmed and validated by immunohistochemical investigation of microglial (Iba-1, CD68), astrocytic (GFAP), and tau (AT8) markers. Findings in cerebral cortex were compared by sex using linear mixed models for PET data and analysis of variance for immunohistochemistry.

**Results:**

Wild-type mice showed an increased TSPO-PET signal over time (female +23%, male +4%), with a significant sex × age interaction (*T* = − 4.171, *p* < 0.001). The Aβ model *App*^*NL-G-F*^ mice also showed a significant sex × age interaction (*T* = − 2.953, *p* = 0.0048), where cortical TSPO-PET values increased by 31% in female *App*^*NL-G-F*^ mice, versus only 6% in the male mice group from 2.5 to 10 months of age. Immunohistochemistry for the microglial markers Iba-1 and CD68 confirmed the TSPO-PET findings in male and female mice aged 10 months. Aβ-PET in the same *App*^*NL-G-F*^ mice indicated no significant sex × age interaction (*T* = 0.425, *p* = 0.673). The P301S tau model showed strong cortical increases of TSPO-PET from 2 to 8.5 months of age (female + 32%, male + 36%), without any significant sex × age interaction (*T* = − 0.671, *p* = 0.504), and no sex differences in Iba-1, CD68, or AT8 immunohistochemistry.

**Conclusion:**

Female mice indicate sex-dependent microglia activation in aging and in response to amyloidosis but not in response to tau pathology. This calls for consideration of sex difference in TSPO-PET studies of microglial activation in mouse models of neurodegeneration and by extension in human studies.

## Introduction

Alzheimer’s disease (AD) is the most prevalent neurodegenerative disease in societies with aging populations [1]. The neuropathology of AD is characterized by the histological triad of accumulation of extracellular amyloid-β peptide (Aβ) plaques, fibrillary tau aggregates within neurons, and the activation of neuroinflammatory pathways mediated by microglia and astrocytes [2–6]. Importantly, the assessment of glial activation by 18-kDa translocator protein (TSPO) positron emission tomography (TSPO-PET) has received growing interest in the last decade [7], which has spurned the development of a wide range of tracers for human studies [8]. Molecular imaging for stratification and monitoring of glial activation could become crucial for target engagement and response assessment of immunomodulatory therapies [9].

Human clinical data indicate that men and women exhibit sex differences in the neuropathological and symptomatic progression of AD [10]. The lifetime risk of later developing AD for 65-year-old females is twice that of men of the same age (12% vs. 6.3%, respectively) [11]. Women also show faster progression from mild cognitive impairment to AD dementia when compared to men [12]. There is growing evidence that sex differences in neuroinflammation pathways including microglia could play a crucial role in driving the sex differences observed in AD [13]. Nonetheless, the few reports on sex differences in TSPO expression have mainly focused on astrocytes in culture. For example, cultured astrocytes derived from female mouse pups showed stronger increases in TSPO mRNA expression upon exposure to bacterial lipopolysaccharide challenge that did cultured astrocytes derived from male littermates [14]. Notably, glial cells express receptors for estrogens and androgens, suggesting the potential for modulation of neuroinflammatory responses by sex steroid hormones [15–17]. Furthermore, a key function attributed to TSPO in microglia is cholesterol transport within the mitochondria [18], which implies downstream effects on the synthesis and metabolism of sex steroid hormones.

A recent human study in cognitively healthy individuals revealed significant sex differences of ^11^C-PBR28 binding in brain, with women showing a higher TSPO-PET signal [17]. However, although TSPO-PET is increasingly used for investigation in vivo of microglial activation in mouse models of AD [19–22], preclinical studies have not previously considered possible sex effects on TSPO-PET findings.

Given this background, we aimed to investigate sex differences in the TSPO-PET signal in wild-type mice as well as in mouse models of Aβ and tau pathology. We tested if the observed effects are independent from potential sex differences in protein aggregation in these models. Finally, we aimed to elucidate if our effects are specific to TSPO-PET binding or if they can be validated by immunostaining for microglia.

## Material and methods

### Animals and study design

All experiments were performed in compliance with the National Guidelines for Animal Protection, Germany, and with the approval of the regional animal committee (Regierung Oberbayern), with oversight by a veterinarian. Mice were housed in a temperature- and humidity-controlled environment with a 12-h light-dark cycle and with free access to food (Sniff, Soest, Germany) and water. Males and females were housed separately in the same animal facility, with cages of equal size and comparable handling. A larger sample of female mice was a priori included in the longitudinal studies listed below due to the higher frequency of aggressive behavior among male cage mates, which can cause space issues when they need to be separated. Cage mates were kept together in small cohorts when entering the imaging facility and no mice had to be separated during longitudinal imaging. All mice with longitudinal imaging data were included in the analysis as stated below. A detailed overview on the sample size of the mouse cohort is provided in Table 1.

**Table 1:** Sample sizes of the mouse cohorts across PET and histo- and immunohistochemistry modalities

Genotype		2–3 M		4–5 M		6–7 M		8–11 M		12–14 M	
Sex		Male	Female	Male	Female	Male	Female	Male	Female	Male	Female
C57BL/6	TSPO-PET	16	31	9	18	2	5	5	27	4	9
	Iba-1									4	4
*App*^*NL-G-F*^	TSPO-PET	6	6	6	15	6	15	6	15		
	Aβ-PET	6	6	6	15	6	15	6	15		
	Iba-1							4	5		
	CD68							4	4		
	Methoxy-x04							4	5		
	GFAP							4	4		
P301S	TSPO-PET	19	33	18	32	15	27	6	6		
	Iba-1							7	7		
	CD68							7	7		
	AT8							7	7		

All PET raw data is derived previous in-house studies conducted using the same tomograph and with identical acquisition parameters. The raw data were reprocessed to obtain maximal agreement of spatial and activity normalization between studies. We included data from descriptive datasets or control groups of therapy/genotype studies:

- *C57BL/6*: Historical cross-sectional ^18^F-GE-180 data from 126 scans of wild-type C57BL/6 mice aged 2–13 months were reanalyzed. Iba-1 staining was performed in four wild-type mice of each sex at 12–14 months of age. In total, 86 of the 126 (68%) TSPO-PET scans of C57BL/6 mice were imaged in parallel with *App*^*NL-G-F*^ and P301S mouse models, whereas the remaining 40 mice (32%) were imaged within the same time-period (2016–2020) but not in parallel with *App*^*NL-G-F*^ and P301S mice included in the current study.
- *App*^*NL-G-F*^*(App*^*NL-G-F/NL-G-F*^): The knock-in mouse model *App*^*NL-G-F*^ carries a mutant amyloid precursor protein (APP) gene encoding the humanized Aβ sequence (G601R, F606Y, and R609H) with three pathogenic mutations, namely Swedish (KM595/596NL), Beyreuther/Iberian (I641F), and Arctic (E618G). Homozygotic *App*^*NL-G-F*^ mice progressively exhibit widespread cerebral Aβ accumulation from 2 months of age [23, 24]. Longitudinal ^18^F-GE-180 data from 75 serial scans of homozygotic *App*^*NL-G-F*^ mice imaged at four different ages (2.5, 5.0, 7.5, and 10 months) from previous [25, 26] and unpublished studies were reprocessed. Six mice of each sex were imaged longitudinally at all four time-points whereas nine females entered PET imaging starting from 5.0 months of age. Only mice with successful imaging until 10 months of age were included. The average cage occupancy (*n* per cage) was 2.1 for females and 1.5 for males. All these mice had a contemporaneous ^18^F-florbetaben Aβ-PET scan, which was reprocessed and analyzed analogously. Iba-1, CD68, GFAP, and methoxy-X04 staining in four to five *App*^*NL-G-F*^ mice of each sex at 11 months of age.
- *P301S*: P301S mice express the human 0N4R tau isoform with the P301S mutation in exon 10 of MAPT gene, which is under control of the murine thy1 promoter on a C57BL/6 background [27, 28]. This model develops aggregates of hyperphosphorylated tau mainly in the brainstem, with onset starting at 2–3 months of age. Tau filaments in these mice appear mostly as half-twisted ribbons, with a lesser abundance of larger paired helical tau filaments resembling those seen in human AD patients. The behavioral phenotype of P301S mice manifests as learning deficits from 2–3 months of age, and onset of motor impairment at 4 months, leading to early death before 12 months of age. Longitudinal ^18^F-GE-180 data from 156 serial scans of P301S mice imaged at up to four different time-points (2, 4, 6, and 8 months) from a previous [21] and an unpublished study were reprocessed. Six mice of each sex were imaged longitudinally at all four time-points whereas 27 female and 13 male mice were imaged from 2 to 6 months of age. Failed imaging sessions at follow-up time-points were excluded. The average cage occupancy (*n* per cage) was 2.6 for females and 1.9 for males. Iba-1, CD68, and AT8 staining had been performed in seven homozygotic P301S mice of each sex at 7–8 months of age.

### PET imaging

#### PET data acquisition, reconstruction, and post-processing

For all PET procedures, including radiochemistry, acquisition and pre-processing, we used an established and standardized protocol [19, 29]. In brief, ^18^F-GE-180 TSPO-PET recordings (average dose 12.3 ± 2.2 MBq) with an emission window of 60–90 min after injection were obtained to measure glial activation. ^18^F-florbetaben Aβ-PET recordings (average dose 11.8 ± 1.9 MBq) with an emission window of 30–60 min after injection were performed for assessment of fibrillar Aβ accumulation. Anesthesia was induced with 3.0% isoflurane delivered via a mask at 3.5 L/min, with maintenance of anesthesia by 1.5% isoflurane during the imaging time window.

#### PET image analysis

We performed all PET data analyses using PMOD (version 3.5; PMOD technologies). Normalization of TSPO-PET emission images was performed by standardized uptake value ratios (SUVR) using previously established reference tissues in the WT (white matter), *App*^*NL-G-F*^ (periaqueductal grey), and P301S (nucleus accumbens) mice [19, 25, 29, 30]. Two bilateral volumes of interest placed in the frontal cortex (comprising 15 mm^3^ each) served for calculation of target-to-reference SUVR. We chose frontal cortex for quantification of TSPO-PET signal based on earlier findings in C57BL/6, *App*^*NL-G-F*^, and P301S mice [21, 25, 30], and due to the documented presence of neuropathology in *App*^*NL-G-F*^ and P301S mice, along with the requirement for a sufficiently large standard volume of interest (VOI).

### Immunohistochemistry

Iba-1, CD68, methoxy-X04, and AT8 immuno- and histochemical stainings were performed as described previously [21, 25]. In brief, we performed a standard free-floating immunofluorescence protocol cortex and brainstem areas matching the PET brain regions. As previously described, perfusion fixed 50-μm-thick brain sections were rinsed either overnight or for 48 h in PBS with 0.2% Triton X-100 containing one of the following primary antibodies: rabbit monoclonal Iba-1 (1:500. Wako: 19-19741), rat monoclonal CD68 (1:500. Bio-Rad: MCA1857), and mouse monoclonal phospho-Tau (1:500. Thermo Fisher: MN1020). After washing in PBS, sections were then incubated in a combination of three secondary antibodies (Alexa 488 goat anti-rabbit, Alexa 594 goat anti-mouse, and Alexa 647 goat anti-rat IgG). Following this step, Aβ fibrils were stained for 25 min upon addition of methoxy-X04 (10 μg/mL in 50% ethanol) at room temperature, followed by washing in buffer (4 times ten min each).

For GFAP staining, free-floating sections were blocked with Normal Goat serum (5%) in PBS + 0.3% Triton X-100 for 1 h at room temperature. We obtained immunohistochemical labelling of astrocytes using the antibody anti-GFAP (Synaptic Systems) 1:500 in 1% NGS blocking solution overnight at 4 °C and the Alexa Fluor 555 secondary antibody anti-rabbit (Invitrogen) 1:500 in 1% NGS blocking solution for 3 h at room temperature. The unbound dye was removed by three washing steps with PBS, and the slices were then mounted on microscope slides with fluorescent mounting medium (Dako, Germany).

We performed quantitation in ImageJ (https://imagej.nih.gov/ij/) with images obtained from a confocal microscope (LSM 780 Axio invers). For each marker, we calculated the percentage coverage of positive staining in a cortical region matching the PET VOI.

### Statistics

In wild-type mice, we first assessed whether there was an age-dependent increase in TSPO-PET signal, using a linear mixed model with age at scanning as a predictor of TSPO-PET controlling for sex (fixed effect) as well as for random slope and intercept. Next, we tested for sex differences in the age dependence of TSPO-PET results by additionally including a sex × age interaction as a predictor.

We applied equivalent models in the *App*^*NL-G-F*^ mice both for TSPO-PET and Aβ-PET, and likewise in the P301S tau transgenic mice for TSPO-PET, to determine whether sex moderated the age-dependent PET increases. When assessing the sex by age interaction effect on the increases in TSPO-PET binding in the *App*^*NL-G-F*^ mice, we additionally controlled the models for individual concomitant Aβ-PET levels to isolate sex-specific effects on the TSPO-PET increases from confound due to the primary fibrillar Aβ pathology.

Effects of sex on histo- and immunohistochemistry area-% readouts were tested by unpaired Student’s *t* test, and effect sizes were calculated as Cohen’s *d*. Statistical analyses were performed in R using the lme4 package for linear mixed models. For all models, we applied an alpha threshold of 0.05 for considering effects to be statistically significant.

## Results

### Stronger elevation of the cortical TSPO-PET signal in females is reproducible in rodents

**Fig. 1:**
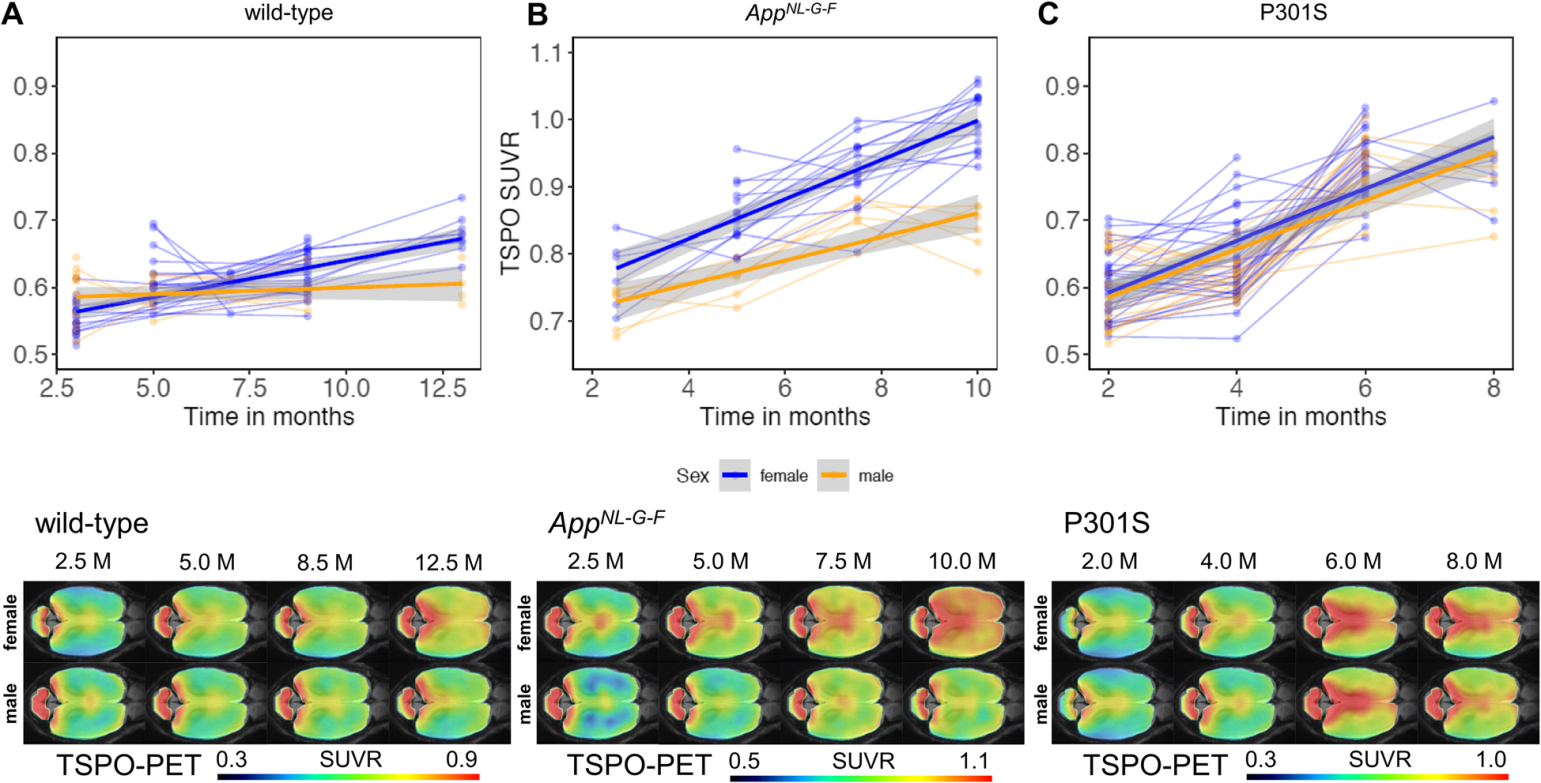
Sex effects on TSPO-PET signal in wild-type mice and mouse models of neurodegenerative diseases. Cortical TSPO-PET quantification is illustrated as a function of age for female (blue) and male (yellow) mice, analyzed by mixed linear models, together with ^18^F-GE-180 TSPO-PET group mean images (*n* = 5–32) at different ages in a horizontal plane projected upon an MRI standard template. Serial scans in the upper part of the figure are indicated by connected data points. 95% confidence intervals are indicated in grey. **a** Female wild-type mice showed a stronger increase of TSPO-PET SUVR than males (*p* < 0.001) from 2.5 to 12.5 months. **b** Female *App*^*NL-G-F*^ mice with Aβ pathology already showed higher SUVR of TSPO-PET at 2.5 months and stronger increases of TSPO-PET SUVR from 2.5 to 10 months as compared to males (*p* = 0.0048). **c** P301S tau transgenic mice showed no significant sex × time interaction on TSPO-PET SUVR with age at time of scanning (*p* = 0.673). TSPO—translocator protein, PET—positron emission tomography, MRI—magnetic resonance imaging, SUVR—standardized uptake value ratio, Aβ—beta amyloid, M—months

**Fig. 2:**
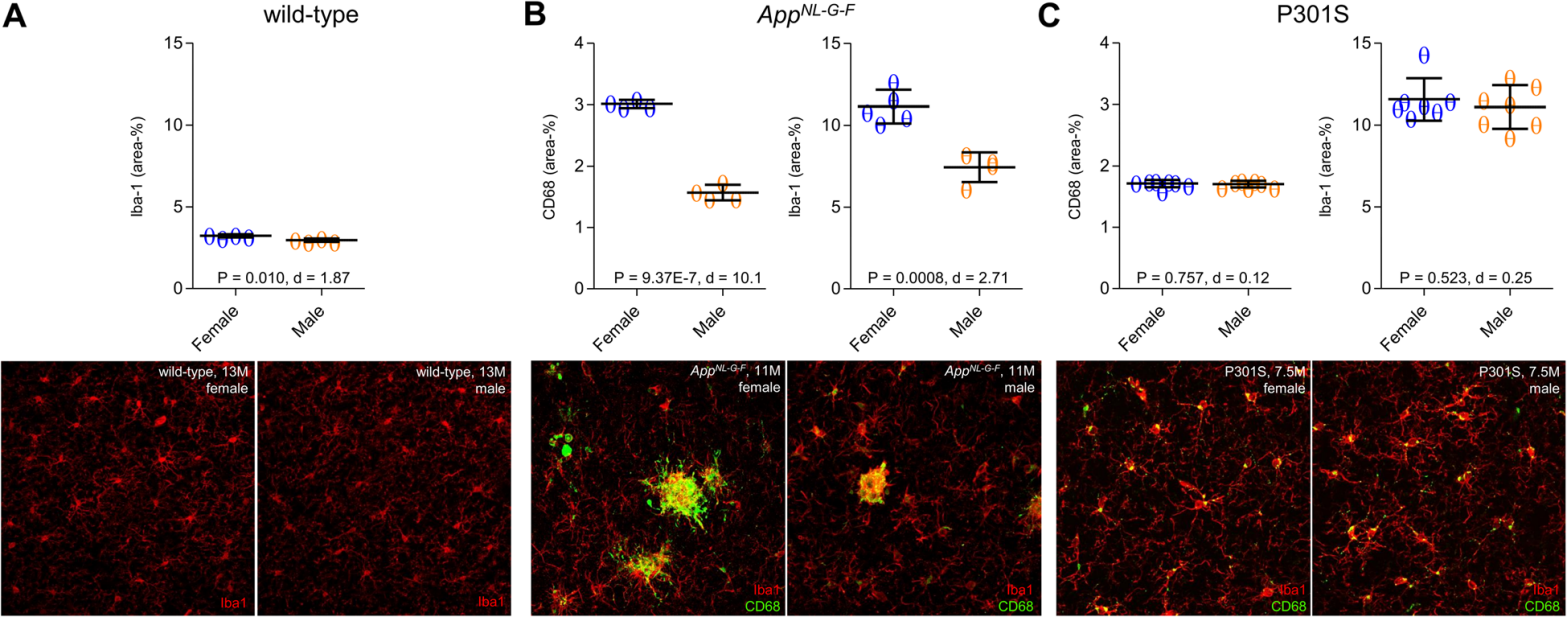
Immunohistochemistry validation. Scatter plots and representative images of Iba-1 and CD68 immunostaining in male and female **a** wild-type, **b** *App*^*NL-G-F*^, and **c** P301S mice

Our first objective was to investigate if recent human findings of higher TSPO-PET signal in cognitively healthy females when compared to males [17] also hold true for healthy wild-type mice. Therefore, we availed ourselves of our large μPET database to make a cross-sectional comparison of ^18^F-GE-180 TSPO-PET scans by sex. Wild-type mice showed increasing TSPO-PET SUVR in cortex with age (*T* = 8.915, b/SE = 0.009/0.001, *p* < 0.001, Cohen’s *d* = 1.608), in line with our earlier report on this phenomenon [30]. Furthermore, we found a significant age × sex interaction (*T* = − 4.171, b/SE = − 0.009/0.002, *p* < 0.001, Cohen’s *d* = 0.755) in these animals, controlling for slope (age) and random intercept, indicating that female mice showed more pronounced TSPO-PET SUVR increases than did male mice (Fig. 1a). Immunohistochemistry confirmed higher Iba-1 quantification in female wild-type mice at 12–14 months of age when compared to males (3.23 ± 0.09% vs. 2.97 ± 0.10%, *p* = 0.010, Cohen’s *d* = 1.87; Fig. 2a). In summary, we confirmed our hypothesis that (as seen in cognitively intact humans) female wild-type mice would show greater age-dependent increases in TSPO-PET binding, which was evident by diverging slopes of female and male mice starting from 6 to 7 months of age.

### Sex-specific TSPO-PET increase in the presence of Aβ pathology

Next, we tested whether female *App*^*NL-G-F*^ mice showed greater longitudinal increases in microglial activation, using TSPO-PET as a proxy. There was a pronounced TSPO-PET increase in the cortex of *App*^*NL-G-F*^ mice with age when considering both female and male mice (*T* = 11.831, b/SE = 0.024/0.002, *p* < 0.0001, Cohen’s *d* = 5.893, linear mixed model, adjusted for sex, random slope, and intercept). We then proceeded to test for a sex × age interaction for TSPO-PET SUVR, controlling for random slope (i.e., age at scanning) and intercept. As hypothesized, we found a significant sex × age interaction for TSPO-PET SUVR (*T* = − 2.953, b/SE = − 0.011/0.004, *p* = 0.0048, Cohen’s *d* = 0.827, Fig. 1b), where the female *App*^*NL-G-F*^ mice showed a greater increase in TSPO-PET SUVR with age than did male mice.

**Fig. 3:**
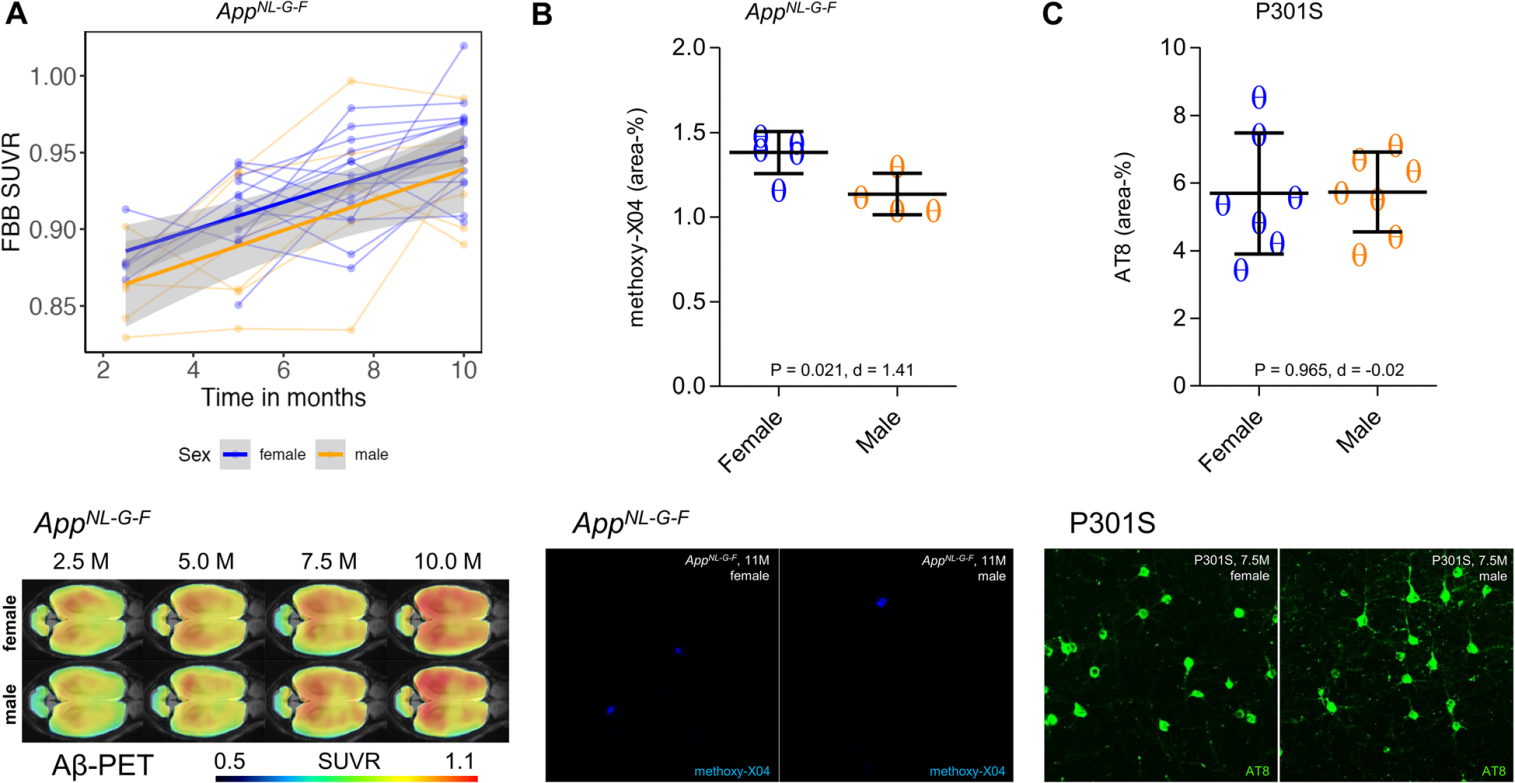
Sex effects on Aβ and tau overexpression in mouse models of neurodegenerative diseases. Cortical Aβ-PET quantification is illustrated as a function of age for female (blue) and male (yellow) mice, analyzed by mixed linear models, together with ^18^F-florbetaben Aβ-PET group mean images at different ages in a horizontal place (*n* = 6–15) projected upon a MRI standard. Serial scans in the upper part of the figure are indicated by connected data points. 95% confidence intervals are indicated in grey. **a** Female and male *App*^*NL-G-F*^ mice showed similar increases of Aβ-PET SUVR from 2.5 to 10 months with a slight offset across the whole observation period. **b** Female *App*^*NL-G-F*^ mice showed slightly higher terminal methoxy-X04 Aβ staining at 11 months when compared to males (*p* = 0.021). **c** P301S tau transgenic mice showed no sex differences in AT8 tau staining at 7–8 months of age (*p* = 0.965). FBB—florbetaben, PET—positron emission tomography, MRI—magnetic resonance imaging, SUVR—standardized uptake value ratio, Aβ—beta amyloid, M—months

**Fig. 4:**
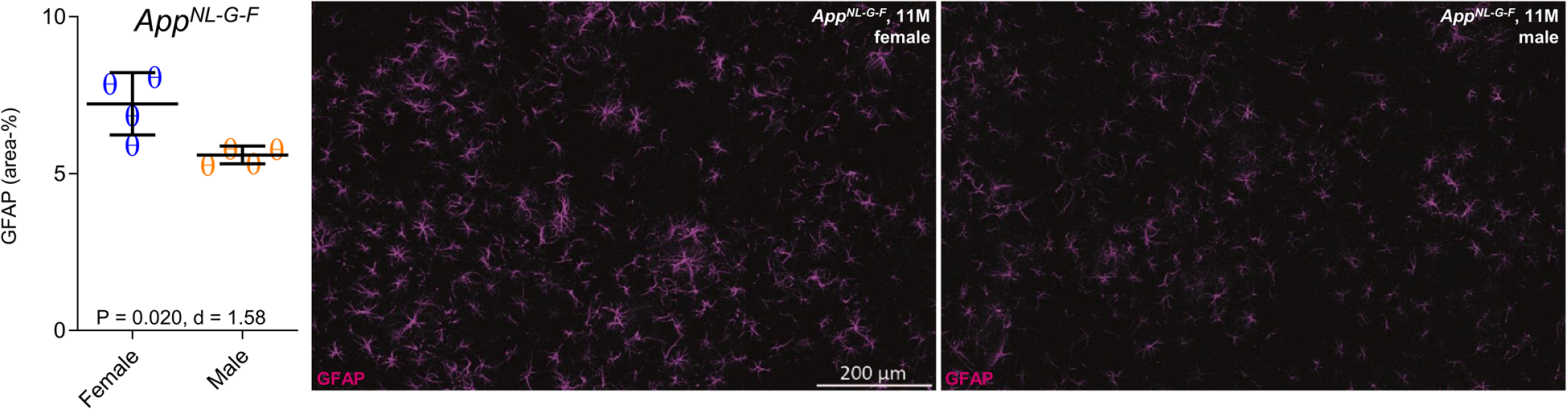
Reactive astrocytes in *App*^*NL-G-F*^ mice. Scatter plots and representative images of GFAP immunostaining in male and female *App*^*NL-G-F*^ mice

To account for potential sex differences in fibrillar Aβ aggregation, we tested whether *App*^*NL-G-F*^ mice showed sex differences in Aβ-PET. Using longitudinal ^18^F-florbetaben-PET data in the same mice, we tested the sex × age interaction on ^18^F-florbetaben-PET SUVR, using linear mixed models controlling for random slope (i.e., age) and intercept. There was no significant sex × age interaction on ^18^F-florbetaben-PET SUVRs (*T* = 0.425, b/SE = 0.001/0.003, *p* = 0.673, Cohen’s *d* = 0.141, Fig. 3a). Thus, we conclude that the longitudinal increases of fibrillar Aβ aggregation in cortex are comparable between male and female *App*^*NL-G-F*^ mice (main effect of age on ^18^F-florbetaben-PET SUVR, controlling for sex, random slope, and intercept: *T* = 6.048, b/SE = 0.009/0.002, *p* < 0.0001, Cohen’s *d* = 1.872). More importantly, the aforementioned sex effect on TSPO-PET remained consistent when controlling for ^18^F-florbetaben-PET SUVR at each time-point (*T* = − 2.926, b/SE = − 0.011/0.0038, *p* = 0.0051, Cohen’s *d* = 0.820), suggesting that sex differences in TSPO-PET SUVR are not driven by sex-specific differences in fibrillar Aβ burden. Immunohistochemistry at the terminal time-point confirmed the observations in vivo, showing higher expression of activated microglial markers in the female mice compared to males (Iba-1: 11.14 ± 1.02% vs. 7.44 ± 0.91%, *p* = 0.0008, Cohen’s *d* = 2.71; CD68: 3.01 ± 0.06% vs. 1.57 ± 0.13%, *p* = 1E−6, Cohen’s *d* = 10.13) (Fig. 2b). Methoxy-X04 histology indicated slightly higher fibrillar Aβ levels in *App*^*NL-G-F*^ females at the terminal time-point (1.38 ± 0.12% vs. 1.14 ± 0.12%, *p* = 0.021, Cohen’s *d* = 1.41, Fig. 3b). GFAP immunohistochemistry showed slightly higher levels of reactive astrocytes in *App*^*NL-G-F*^ females at the terminal time-point (7.23 ± 1.00% vs. 5.60 ± 0.28%, *p* = 0.020, Cohen’s *d* = 1.58, Fig. 4).

In summary, we find a striking effect of sex on the age-dependent increase in cortical TSPO-PET signal in Aβ-transgenic mice, in the absence of notable sex differences in fibrillar Aβ pathology to ^18^F-florbetaben-PET SUVR, and with only slightly higher fibrillar Aβ to histology at the terminal time-point. This indicates that the sex difference in microglial activation is not readily attributable to sex differences of fibrillar Aβ pathology.

### TSPO-PET increases in response to tau pathology are not moderated by sex

Next, we tested whether sex-specific increases of the TSPO-PET signal were specifically related to cerebral Aβ accumulation, or rather a more general function of AD-like brain pathology. To this end, we assessed longitudinal TSPO-PET in P301S tau transgenic mice, finding the expected longitudinal increases in TSPO-PET SUVR with age (*T* = 15.77, b/SE = 0.038/0.002, *p* < 0.0001, Cohen’s *d* = 2.987). There was, however, no significant sex × age interaction with TSPO-PET SUVR, controlling for slope (i.e., age) and random intercept (*T* = − 0.671, b/SE = − 0.003/0.005, *p* = 0.504, Cohen’s *d* = 0.128, Fig. 1c). Thus, the TSPO-PET SUVR increases in response to 4-repeat isoform tau pathology did not differ between male and female P301S mice. Terminal immunohistochemical analysis likewise did not indicate any significant sex differences in microglial markers (Iba-1, 11.56 ± 1.29% vs. 11.10 ± 1.33%, *p* = 0.523, Cohen’s *d* = 0.25; CD68, 1.72 ± 0.06% vs. 1.71 ± 0.05%, *p* = 0.757, Cohen’s *d* = 0.12; Fig. 2c) or AT8-positive tau accumulation (5.69 ± 1.79% vs. 5.73 ± 1.18%, *p* = 0.965, Cohen’s *d* = -0.02; Fig. 3c) in P301S mice aged 7–8 months.

## Discussion

This is the first investigation aiming to elucidate sex differences in microglial activation in aging wild-type mice and mouse models of AD-like pathologies. Our data show a sex-specific effect on the cortical TSPO-PET signal and immunohistochemical markers of microgliosis in wild-type mice, with a greater age-dependent increase in female mice, thus in line with human TSPO-PET data in cognitively healthy men and women. Notably, we also observed a strong sex-specific effect on TSPO-PET signal during accumulation of Aβ pathology, indicating higher TSPO-PET binding in female *App*^*NL-G-F*^ mice than in males. Contemporaneous Aβ-PET from the same mice allowed us to adjust TSPO-PET results for possible confounding effects due to sex differences in the progression of fibrillar Aβ pathology. In contrast, P301S mice did not indicate sex differences in TSPO-PET signal increases in response to their accumulation of tau pathology. Terminal histo- and immunohistochemical analyses of phosphorylated tau, Aβ, and microglial markers confirmed all of the main PET findings.

Similar to a recent multi-center TSPO-PET in cognitively healthy men and women [17], we observed a strong effect of sex on the cortical TSPO-PET signal in a cross-sectional cohort of wild-type mice, indicating progressively higher TSPO-PET signal in cortex of females as compared to males. Importantly, we used data from mice housed under standard conditions, and with a completely matched TSPO-PET protocol, thus minimizing variance from methodological and environmental factors. This is critically important, given our compilation of data from various studies.

The human TSPO-PET study showed a more distinctly higher grey matter signal in younger women, which declined with increasing age of the subjects [17]. In contrast, our wild-type mouse data showed a sex difference in TSPO-PET favoring the females, which became more prominent with increasing age. However, we note that our tracking of TSPO-PET extended to only to 13 months of age, which is perhaps comparable to middle age in humans [31]. Therefore, sex differences in TSPO-PET may follow differing time-courses in healthy mice and humans in relation to their vastly different lifespans. Our histo- and immunohistochemical validations of the in vivo TSPO-PET results present a distinct advantage of our preclinical study. Thus, we unambiguously establish the congruence of TSPO-PET findings with greater elevation of the microglia marker Iba-1 in aged female wild-type mice when compared to males. This result also supports the claims made in the human study cited above that increasing TSPO-PET binding with healthy aging is attributable to microglial activation, i.e., neuroinflammation. The large variability of human TSPO-PET binding in healthy individuals [17] and the allelic dependence of most TSPO tracers [8] predict a need for substantial group sizes to detect sex interactions with TSPO-PET signal in neurodegenerative diseases like AD. Our present use of inbred mouse lineages may have tended to reduce variance in the various endpoints, and our use of transgenic mouse models with overexpression of Aβ or hyperphosphorylated tau allows the isolation of effects of different protein aggregates on the interaction between sex and TSPO-PET expression. While preclinical AD patients can show Aβ-positivity and tau-negativity [32], there is lingering uncertainty about the threshold of disease progression for detection of tau in cerebrospinal fluid or tau-PET assessments [33]. However, our investigation reveals a sex-specific effect on TSPO-PET increases during accumulation of Aβ pathology in *App*^*NL-G-F*^ mice, with an interaction of a magnitude similar to that seen in aging wild-type mice. The progression of Aβ pathology is more rapid in female AD-model mice than in males [34]. A primary accumulation of fibrillar Aβ triggers a secondary neuroinflammation mediated by activated microglia [35]. Preliminary results had indicated sex differences in Aβ accumulation in *App*^*NL-G-F*^ mice, albeit with onset in the interval between 12 and 18 months [23], thus occurring after the present observation period. We did not observe sex differences of the Aβ-PET increase in *App*^*NL-G-F*^ mice and inclusion of Aβ-PET results in the linear mixed model did not alter the sex difference in TSPO-PET. Methoxy-X04 staining indicated moderately higher fibrillar Aβ pathology in female *App*^*NL-G-F*^ mice compared to male *App*^*NL-G-F*^ mice, which may reflect greater sensitivity of the histological staining [20], or the preponderant weighting of the Aβ-PET signal to fibrillar Aβ and its insensitivity to soluble Aβ proportions. Thus, we cannot exclude the possibility that the effect of sex on TSPO-PET in *App*^*NL-G-F*^ mice may be influenced by sex differences in the biochemical nature of the Aβ pathology. While the main attribution of the TSPO-PET signal is to activate microglia, there is also a contribution from pro-inflammatory astrocytes [36]. We note that the proportion of astroglial versus microglial contributions to TSPO-PET signals might differ between male and female mice and our immunohistochemistry data showed sex differences for both CD68 reactivity and GFAP reactivity in *App*^*NL-G-F*^ mice. A direct correlation analyses between TSPO-PET and immunohistochemistry in larger cohorts of male and female *App*^*NL-G-F*^ mice could better substantiate the cellular origin of the TSPO-PET signal.

Surprisingly, we were unable to detect corresponding sex-specific effects on longitudinal TSPO-PET increases in the P301S tau mouse model. Negative PET findings are supported by the absence of sex differences in Iba-1 and CD68 immunohistochemistry, and by the equal levels of hyperphosphorylated AT8-positive tau in female and male P301S mice at 7 months of age. Although there were significant sex differences in microglial microRNA expression model mice [37], our present in vivo findings do not indicate any sex difference in the TSPO-PET binding with aging of P301S mice. Indeed, these tauopathy model mice did not manifest the sex effect seen in wild-type mice, perhaps due to masking by effects of strong tau pathology in aging P301S mice. We also note that PET imaging paradigms give a macroscopic view of neuroinflammation components in mouse brain as compared to immunohistochemistry or in situ hybridization methods. However, we emphasize the translational relevance of neuroinflammation PET biomarkers [6]. Thus, the synthesis of our combined findings comprise strong evidence for a sex-specific effect on the TSPO-PET signal in aging wild-type mice, and the association between microglia activation with Aβ accumulation in transgenic mice, without corresponding association with tauopathy in P301S mice. Present observations are hypothesis generating for studies of microglial activation by manipulation of sex hormones, i.e., by ovariectomy or orchidectomy [10]. Furthermore, cross-breeding of Aβ and tauopathy mouse models with TSPO knock-out mice [38] could mechanistically help elucidate the effect of sex differences of TSPO on functionally related pathways such as cholesterol synthesis. We note the present imbalance of numbers of male and female mice, which arises from retrospective design of this study.

There is growing evidence that tau pathology follows accumulation of Aβ in AD patients [39]. Recent data showed that NLRP3 inflammasome activation triggers tau pathology [40], and importantly, there is evidence that women present a higher level of tau pathology even at the preclinical stage of AD [41]. This stands in contrast to present finding of absent sex differences in tauopathy in the P301S model mice, which lack Aβ pathology. Thus, enhanced microglial activation in response to Aβ could constitute the key factor resulting elevated in tau pathology in female AD patients. TSPO-PET in conjunction with tau- and/or Aβ-PET in cohorts of AD and primary tauopathy patients (i.e., progressive supranuclear palsy or corticobasal degeneration) would translate our present findings into the study of human disease.

## Conclusions

Sex-specific effects on the age-dependent increase in cortical TSPO-PET signal are present in wild-type mice and in mouse models of neurodegenerative diseases. Sex moderates the cortical microgliosis in response to amyloidosis but not to tau pathology in transgenic mice. Strict controlling for sex is required for TSPO-PET studies of microglial activation in mouse models of neurodegenerative diseases and requires attention in corresponding studies in human disease.

## Data Availability

The datasets used and/or analyzed during the current study are available from the corresponding author upon reasonable request.

## Abbreviations

Aβ: Beta amyloid
AD: Alzheimer’s disease
ANOVA: Analysis of variance
APP: Amyloid precursor protein
B/SE: Main and interaction effects
IHC: Immunohistochemistry
MRI: Magnetic resonance imaging
PET: Positron emission tomography
M: Months
MAPT: Microtubule-associated protein tau
MCI: Mild cognitive impairment
SPM: Statistical parametric mapping
SUV: Standardized uptake value
SUVR: SUV ratio
TSPO: Translocator protein
VOIs: Volumes of interest
WT: Wild-type
